# Some Principles of Treatment of Gunshot Wounds

**Published:** 1919-01

**Authors:** 


***Some Principles of Treatment of Gunshot Wounds.*** By CAPTAIN C. H. UPCOTT. *American Journal of Sciences*, August, 1918.

There is one common point where after four years' experience all surgeons seem to agree : the need for free enlargement of the wounds. The author's experience has been acquired in a casualty clearing station; the patients arrive there from 6 to 24 hours after receipt of their wounds, and generally in a most exhausted condition. Transfusion of whole blood, subcutaneous or rectal infusions of saline or glucose, and hypodermic injections of camphorated oil often prove very useful stimulants.

The following points are of importance in the first treatment of wounds : first, the immediate destructive effect of the projectile is not strictly limited to its path; second, every wound is practically permeated with foreign material bearing aerobic and anaerobic organisms; hence the necessity of having widely open wounds from whose surfaces all foreign matter and dead tissues are removed.

*Types of Wounds.* These may be roughly classified as follows :

(1) Simple perforating wounds in which the track is of about the same diameter as the skin aperture. They generally require nothing more than excision of skin and fascia.

(2) Wounds in which the destruction of skin and superficial tissue is of greater extent than the destruction of deeper tissues. After complete excision of all damaged tissues primary suture may be performed.

(3) Wounds in which the skin aperture is small in relation to the extent of damage inflicted on deeper structures. These divide into (*a*) lodging wounds, and (*b*) traversing wounds.

TECHNIC.

*Sterilization of the Skin.* The skin should be rubbed at first for 2 or 3 minutes with swabs wet with Dakin's sol. or eusol, then with methylated spirit for one minute. Finally it should be painted with picric solution. The skin treated thus can be rendered sterile.

*Excision of Gutter Wounds.* An elliptical incision through skin and fascia, until beneath the deepest part of the wound, should be practised one fourth inch from the edges of the wound, cutting out a wedge of tissue enclosing the wound and not opening into it at any part. All bleeding must be carefully stopped and the wound sutured with silk-worm gut. Gum-mastic varnish, or better " aeroplane dope ", makes a good dressing. If the wound is opened into at any part during the operation, primary suture should not be resorted to. The wound should be left wide open and the Carrel-Dakin treatment instituted.

*Excision of Traversing Wound with Explosive Exit.* Make a cone-shaped incision of the wound, 1/4 inch from its edges. Dressing may be a small central tube to the track of the missile surrounded by salt pack or Carrel-Dakin.

*Tunnel Wounds.* Exit and entrance wounds should be connected by an incision and then excised like gutter wounds.

*Traversing Shell Wounds.* Entry and exit should be excised by elliptical incisions and the tissues around the wound excised to the depth of the knife blade in one piece. The crushed muscles and aponeurosis in the deeper parts should be removed by means of forceps.

Powerful retraction must be avoided by the use of free incisions, gas infection always developing in dead or dying tissues, and therefore the following remarks are of importance.

(1) If a muscle is deprived of its blood supply, it will not bleed when cut and will probably die.

(2) A dead muscle will neither contract nor bleed when cut.

(3) A muscle in the first stage of invasion by anaerobes loses its normal resilience, and has a peculiar brick-red color.

(4) In the later stages of invasion the muscles become crepitant and exude a dark reddish-brown foul-smelling fluid.

*Lodging Shell Wounds.* These wounds should be treated like the former ones after the projectile has been removed.

*Hemostasis.* Great care should be devoted to hemostasis; pools of blood accumulating in the corners of the wound favor the progress of sepsis.

*Local Rest* is supplied by firm dressings and suitable splints.

*After-Treatment.* The salt pack should be considered as the best dressing in times of pressure, as long as it is carefully applied.

